# Identification and genetic diversity of hepatitis E virus in domestic swine from Slovakia

**DOI:** 10.1186/s12917-021-02936-4

**Published:** 2021-06-30

**Authors:** Anna Jackova, Katarina Dudasova, Slavomira Salamunova, Rene Mandelik, Jaroslav Novotny, Stefan Vilcek

**Affiliations:** 1 Department of Epizootiology, Parasitology and Protection of One Health, University of Veterinary Medicine and Pharmacy, Komenskeho 73, 041 81 Kosice, Slovakia; 2 Clinic of Swine, University of Veterinary Medicine and Pharmacy, Komenskeho 73, 041 81 Kosice, Slovakia

**Keywords:** Hepatitis E virus, Pig, Slovakia, Phylogenetic analysis, Novel subtype

## Abstract

**Background:**

Hepatitis E virus (HEV) is agent causing hepatitis worldwide. Originally considered to be limited to developing countries, this virus was also detected in developed countries. In recent years an increasing number of reports indicate that farmed domestic pigs are widely infected with HEV in several European countries. The HEV status in Slovakia is still missing.

**Results:**

In this study, the circulation of HEV among domestic swine in Slovakia and genetic diversity of the virus was studied. Overall HEV RNA was detected in 53/388 (13.7, 95% CI: 10.40–17.48%) pig rectal swabs in five production stages (age categories) with statistically significant differences among all the stages. The highest HEV prevalence was observed in weaners 24/81 (29.6, 95% CI: 19.99–40.81%) and then significantly declined in growers and fatteners. No HEV was detected in suckling piglets and sows. Twenty-eight partial sequences of ORF1 (242 bp) and seventeen of ORF2 (304 bp) were analysed. Phylogenetic analysis and *p*-distance comparisons confirmed in both ORFs that all Slovak HEV sequences belong to the genotype HEV-3, major clade 3abchij with higher identity to 3a and 3i subtypes. Three sequences were outside of all lastly updated HEV-3 subtypes.

**Conclusion:**

This is the first report to fill the information gap about HEV infection in pigs in Slovakia. The results suggested a lower prevalence of HEV in Slovak pig farms than observed in other European countries. While most HEV isolates were typed as HEV-3 clade 3abchij, three sequences were unclassified.

## Background

Hepatitis E virus (HEV) is one of the most common agent causing hepatitis worldwide. Originally considered to be limited to developing countries, it has recently also been shown to be widespread in developed countries [1, 2]. A recent surveillance report of hepatitis E virus infection in the European Union/European Economic Area (EU/EEA) countries showed a tenfold increase between 2005 and 2015 [3].

Hepatitis E virus is a small virus with the diameter of 27–32 nm, generally considered to be non-enveloped [2]. The HEV genome consists of a single-stranded positive sense RNA with a size of approximately 7.2 kb containing three open reading frames (ORFs) and short untranslated regions. The ORF1 encodes non-structural proteins, ORF2 encodes the glycoprotein that forms the viral capsid and ORF3 encodes a small multifunctional protein involved in virus morphogenesis and release [4]. HEV, a highly variable virus, belongs to the family *Hepeviridae,* genus *Orthohepevirus A* which is subdivided into seven genotypes. Genotypes HEV-1 and HEV-2 are restricted to humans. Genotype HEV-3 was detected by Meng et al. [5] in domestic pigs for the first time in the USA and subsequently over the world in different animal species (e.g. wild boar, mongoose and deer) and humans. Similarly, members of genotype HEV-4 have been detected in both animals and humans. On this basis HEV-3 and HEV-4 are considered to be zoonotic [6, 7]. The genotypes HEV-5 and HEV-6 have been found only in Japanese wild boar [8]. The genotype HEV-7 has been isolated in dromedary camels and is also considered to be zoonotic [9].

The HEV genotypes have been subdivided into numerous subtypes. Subdivision into 24 subtypes has been proposed by Lu et al. [10], but classification of HEV subtypes is still under discussion. The inconsistencies concerning the subtype classification were demonstrated using different methodological approaches [11, 12]. Subsequently, Smith et al. [13] proposed reference sequences for HEV subtypes to improve communication between researchers and to help to clarify the epidemiology of this important pathogen. The use of common reference sequences for each subtype today assists with the interpretation of epidemiological and evolutionary studies of HEV. HEV-3 subtypes 3a, 3b, 3c, 3 h, 3i, 3j form one major clade-3abchij, while subtypes 3e, 3f and 3 g form another clade-3efg [14]. These two clades correspond to the previously named groups 3-I and 3-II [15], groups 3.1. and 3.2 [11]. and group 1 and 2 [16]. The values of nucleotide *p*-distances among HEV genotypes and subtypes show a complex pattern with multiple hierarchies of relatedness [13].

Several studies have confirmed that domestic swine are suspected to be one of the main reservoirs of HEV [7, 17]. In recent years an increasing number of reports indicate that farmed domestic pigs are widely infected with HEV in several European countries [18–22]. The prevalence and transmission of HEV in domestic swine population in Western European countries described by Berto et al. [22] confirmed that HEV circulated in production stages from weaners to fatteners. The study on Spanish pig farms showed that circulation of HEV started in pigs of 8–9 weeks old [18, 19]. HEV infection in pig farms of Northern Italy was more often detected in weaners as well [20]. The occurrence of HEV in Czech and Hungarian pig farms varied in the range 22.7–39% with highest detection of virus among 11–16 week-old pigs [23–26]. The HEV status in Slovakia, an EU member country geographically lying in Central Europe, is still missing.

The aim of this study was to gain the first insight into the circulation of HEV in Slovak domestic swine at different stages of production. Molecular-genetic analysis of nucleotide sequences was focused on the typing of HEV isolates to extend our knowledge of genetic variability of the virus.

## Results

### Detection of HEV RNA in different production stages

HEV positive samples were detected in 13 of 25 investigated pig farms. The farm size did not affect the HEV prevalence. Overall, 53 out of 388 (13.7, 95% CI: 10.40–17.48%) rectal swabs were tested positive for HEV RNA (Table 1). No positive sample was detected in the suckling piglets (≤ 4 weeks) or the sows. The circulation of HEV started in production stage of weaners (5–10 weeks) in which the highest HEV presence (24/81–29.6, 95% CI: 19.99–40.81%) was observed. Significantly lower numbers of positive samples (*P* < 0.05, χ2 = 6.746) were detected in older age category of growers (10/67–14.9%; 95 CI: 7.40–25.74%). At the same time significant decrease of positive samples (*P* < 0.01, χ2 = 10.807) was observed in fatteners (19/135–14.1%; 95% CI: 8.69–21.10%) as well. Differences in HEV infection among all five production stages were statistically significant (*P* < 0.01, χ2 = 28,444).

**Table 1:** Detection of HEV RNA in domestic swine of different production stages

Production stage (age in weeks/years)	Number of farms	Average number of samples per farm	Total number of samples	HEV+ n (%)	95% CI
Suckling piglets (≤ 4 weeks)	7	7–8	53	0 (0%)	0.00–6.72
Weaners (5–10 weeks)	14	5–6	81	24 (29.6%)	19.99–40.81
Growers (11–16 weeks)	9	7–8	67	10 (14.9%)	7.40–25.74
Fatteners (≥ 17 weeks)	16	8–9	135	19 (14.1%)	8.69–21.10
Sows (1–3 years)	7	7–8	52	0 (0%)	0.00–6.85
Total			388	53 (13.7%)	10.40–17.48

### Sequences and phylogenetic analysis

Twenty-eight PCR products from the ORF1 gene (242 bp) and seventeen from the ORF2 gene (304 bp) were sequenced. They were compared to sequences from the NCBI GenBank database representing swine HEV strains belonging to the reference sequences of genotypes and subtypes proposed by Smith et al. [13].

**Fig. 1:**
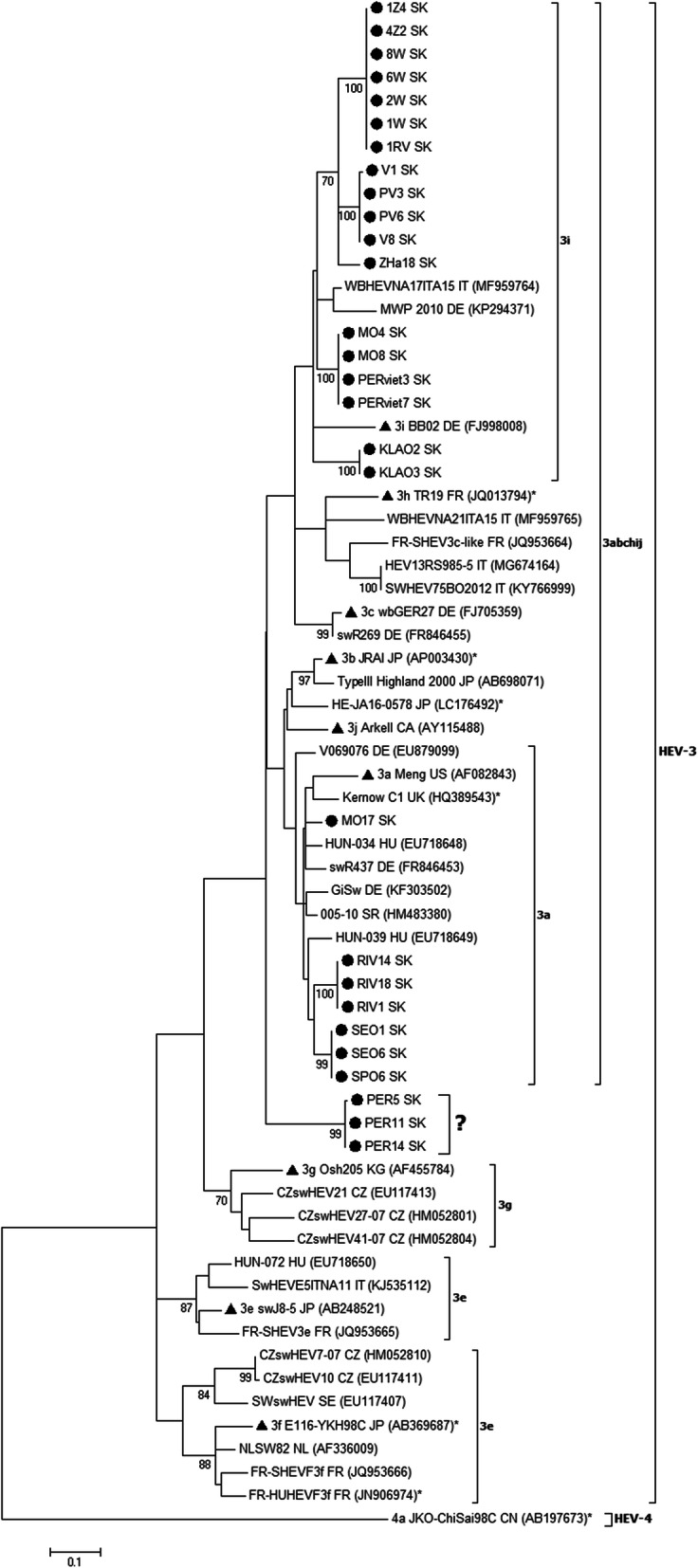
Phylogenetic tree of HEV nucleotide sequences based on partial ORF1 (242 nt). The maximum likelihood phylogenetic tree was built with GTR + G + I substitution model and a bootstrap resampling process (1000 replications) was used to assess node support. Bootstrap values > 70 are indicated at their respective nodes. The tree included 66 HEV sequences: i) 28 Slovak HEV sequences are indicated by a black circle ii) 38 HEV sequences were selected from NCBI GenBank database, HEV-3 reference subtypes according Smith et al. [13] are indicated by a black triangle. Highly diverse sequences outside known subtypes are marked with a question mark. The HEV-4 strain was used as an outgroup. All sequences are denoted by name sequences, ISO code country of origin and NCBI GenBank accession number in brackets. Human HEV sequences are marked with an asterix. The scale bar indicates nucleotide substitutions per site

The phylogenetic analysis based on the partial ORF1 gene revealed that all Slovak HEV sequences were clustered within the genotype HEV-3 with separate branches according to the farm of origin (Fig. 1). Four HEV sequences (MO17, SEO1, SEO6, SPO6) clustered unambiguously into the 3a subtype with nucleotide *p*-distances ranging from 0.085 to 0.106 to the 3a reference strain. Three sequences RIV1, RIV14, and RIV18 showed higher *p*-distances (0.123) to the 3a reference strain. Other analysed sequences could be classified into the major clade 3abchij with ambiguous classification into subtypes, except for three remarkable sequences. These three sequences (PER5, PER11, PER14) clustered in the phylogenetic tree outside of both major clades (3abchij and 3efg) of the HEV-3 genotype (labelled with a question mark) showing high *p*-distance values 0.140–0.178 to the reference strains of all HEV-3 subtypes.

**Fig. 2:**
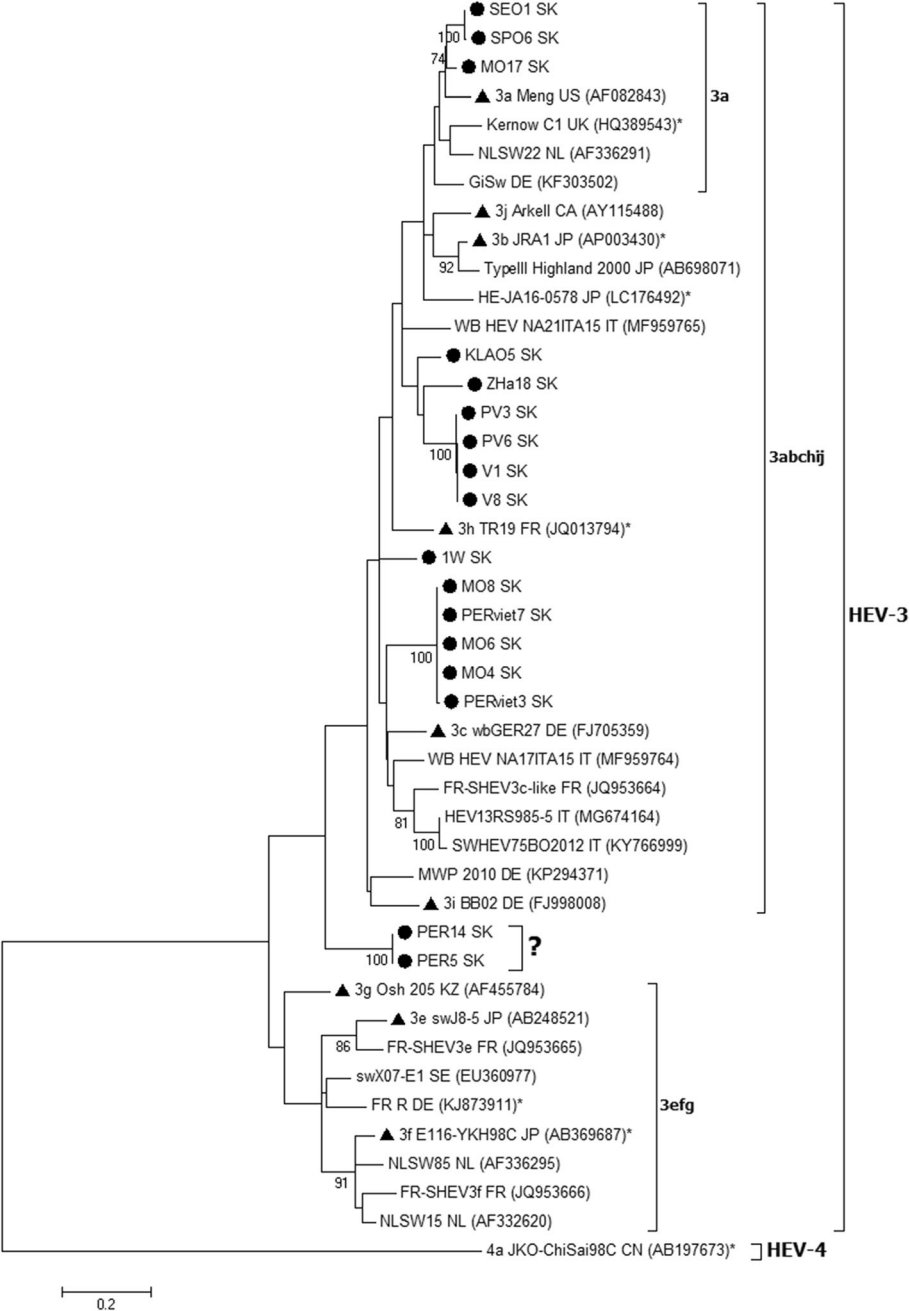
Phylogenetic tree of HEV nucleotide sequences based on partial ORF2 (304 nt). The maximum likelihood phylogenetic tree was built with K2 + G + I substitution model and a bootstrap resampling process (1000 replications) was used to assess node support. Bootstrap values > 70 are indicated at their respective nodes. The tree included 44 HEV sequences: i) 17 Slovak HEV sequences are indicated by a black circle ii) 27 HEV sequences were selected from NCBI GenBank database; HEV-3 reference subtypes according Smith et al. [13] are indicated by a black triangle. Highly diverse sequences outside known subtypes are marked with a question mark. The HEV-4 strain was used as an outgroup. All sequences are denoted by name sequences, ISO code country of origin and NCBI GenBank accession number in brackets. Human HEV sequences are marked with an asterix. The scale bar indicates nucleotide substitutions per site

The analysis of the ORF2 sequences supported results achieved with ORF1. The phylogenetic and sequence analysis of the partial ORF2 (Fig. 2) confirmed the clustering and subtyping of Slovak HEV sequences with ORF1.

## Discussion

In this work we describe for the first time the detection and genetic characterization of HEV in Slovakia. The 13.7% overall percentage of HEV positive samples found in Slovakian pig farms was less than 18.6% detected in pigs from three different Italian provinces [27], or 18.8 and 23.3% found in Spanish pigs [28, 29] and 21% detected in Hungarian pig farms [25]. Almost similar results were found in Portugal and Estonia [22, 30]. High rate of virus shedding (42%) were also found in farms from Northern Italy [20]. A significant increase of HEV prevalence from 22% in 1999 to 55% in 2007 was reported for Dutch pig farms [31].

In our study, the presence of HEV depended on the age of the pigs. No HEV was detected in the youngest category of suckling piglets (≤ 4 weeks). The occurrence of HEV started in pigs of 5–8 weeks old. The same results reported from other laboratories [19, 31]. De Deus et al. [18] detected HEV first time in 9 weeks old pigs. The absence of HEV in suckling piglets might be explained by protection with specific maternal antibodies [5] although some authors detected HEV in suckling piglets ranging from 9 to 11.8% [25, 29, 32]. We detected the highest percentage of HEV (29.6%) in weaners with a significant decrease in growers and fatteners. A higher prevalence of HEV in weaners is consistent with the results of Berto et al. [22] who observed 32% of HEV in weaners in Portugal industrial farms. The highest HEV prevalence (41 and 52.9%) in weaners was also reported by Fernandez-Barredo et al. [29] and Leblanc et al. [32].

Weaners are the most critical category of animals, because declining maternal antibodies results in weaker immunity and the highest susceptibility to infections, including HEV [5]. Immunity is stronger in older animals and it corresponds with our data on lower detection of HEV in growers and fatteners. Very similar observation of HEV in adult pigs older than 6 months has been reported by other authors [29, 32]. A significant decline of HEV detection has been described in adult domestic pigs (> 6 months) in Corsica, a French region hyper-endemic for HEV [33]. A strong decline of HEV from 100% in weaners to 40% in growers and 0% in fatteners observed by Seminati et al. [19] in Spain is in agreement with the trend observed in our work. No doubt, despite the lower prevalence of HEV in older pigs, many pigs at slaughter age are infected with HEV*.* It should be emphasized that slaughtered HEV infected pigs are a significant risk factor for the introduction of virus to the food chain [34] and represent a risk of foodborne transmission of HEV to the human population [35]*.*

HEV is not always detected in sows as we demonstrated in this work and as has been reported for several Spanish pig farms [19]. A very low HEV prevalence in sows was also detected in Italy and United Kingdom [27, 36]. On the other hand, 53.4% HEV prevalence in old sows (1–5 years) and 38.6% in young sows (11–15 months) was reported in Italy [20]. Pig farms with a high HEV prevalence may in future have difficulty sharing their sows with other farms.

The sequencing of partial fragments from the ORF1 and ORF2 regions revealed genetic diversity and enabled typing of viral isolates. The phylogenetic analysis in both genomic regions (Figs. 1 and 2) indicated that all Slovak HEV sequences were clustered within the genotype HEV-3 and formed separate clades according to their origin. Most European pig HEV isolates are usually found in this genotype along with several human isolates.

Within the HEV-3 genotype it is difficult to divide sequences into subtypes. At present, there are several combined criteria for this typing. The classification into HEV-3 subtypes is based on the comparison with the reference strains and the value of nucleotide *p*-distances (<0.123) within subtypes as proposed by Smith et al. [13]. In addition, the location of sequences in the phylogenetic tree also assist with the typing of HEV-3 isolates.

Looking closer at the phylogenetic tree prepared from ORF1 sequences (Fig. 1) it is clear that Slovak HEV-3 nucleotide sequences were grouped within a mixed 3abchij cluster. The comparison based on nucleotide *p*-distances (0.085–0.106) showed that four HEV sequences from three farms (MO17, SEO1, SEO6, SPO6) unambiguously belong to the 3a subtype together with the reference strain 3a Meng (AF082843). Three sequences RIV1, RIV14, RIV18 showed *p*-distances 0.123, which is close to the limit for a subtype. They were located in the clade with reference strain 3a.

Six Slovak HEV-3 sequences (MO4, MO8, PERviet3, PERviet7 and KLAO2, KLAO3) belonged within the 3i subtype (see 3i cluster on Fig. 1). Their typing was based not only on their position in the phylogenetic tree but also on the nucleotide *p*-distances (0.119) with the reference strain 3i BB02 (FJ998008). This conclusion was supported by comparison with the Italian strain WBHEVNA17ITA15I (MF959764) which has been recently confirmed as subtype 3i by De Sabato et al. [37]. When comparing six Slovak sequences to this strain, the low nucleotide *p*-distances (0.076–0.106) also support their typing into 3i subtype. However, to be correct, this conclusion is disturbed by the observation that *p*-distance of the discussed six Slovak sequences is in the range from 0.110 to 0.114 with the reference strain wbGER27 (FJ705359) for subtype 3c, as well.

Another 12 Slovak sequences (see top of the tree – Fig. 1) compared with the Italian 3i strain mentioned (MF959764) showed similar low *p*-distances in the range from 0.097 to 0.106. This data together with clustering in the maximum likelihood phylogenetic tree (Fig. 1) support a conclusion that they belong to the subtype 3i.

Most HEV sequences from Western European countries such as the Netherlands, France, Italy, Spain and Sweden are clustered into subtypes 3e, 3f and 3 g (3efg major clade) [15, 17, 21, 22]. Surprisingly, none of Slovak HEV sequences clustered into 3efg clade despite all HEV isolates from the neighbouring country Czech Republic published so far have fallen into subtypes 3f and 3 g [23, 24]. When looking at HEV isolates from another neighbouring country Hungary they have been clustered into the subtypes 3e and 3a [25], indicating a possible relationship to 3a isolates from Slovakia, most probably due to the common trade of animals.

The last three Slovak HEV sequences (PER5, PER11, PER14 – Fig. 1, marked with a question mark) originating from a farm in Eastern Slovakia are highly divergent and clustered outside of both major 3abchij and 3efg phylogenetic clades. These sequences showed relatively high nucleotide distances (*p*-distances 0.140–0.178) when compared to *p* < 0.123 defined by Smith et al. [13] for the subtype. They were unclassified to any subtype in our study.

The analysis of HEV-3 sequences in ORF2 (Fig. 2) confirmed results from ORF1 for the subtype 3a (*p*-distances 0.070–0.083). The clustering of other sequences into HEV-3 subtypes was not unambiguous (3i and 3c with *p*-distances 0.120–0.123) but the classification into major clade 3abchij was confirmed as with ORF1. Similar as in ORF1 the sequences PER5 and PER14 were located outside of clades 3abchij and 3efg with the *p*-distance values of 0.147–0.187.

## Conclusion

This is the first report to fill the gap about HEV infection in pigs in Slovakia. The results indicated a lower prevalence of HEV in Slovak pig farms than observed in other European countries. It is promising that HEV did not circulate on all farms, nor in all production categories. Nevertheless, the occurrence of HEV in fatteners (slaughter age) is a risk to public health, because HEV is considered to be a foodborne pathogen with potential zoonotic transmission. While most HEV isolates were typed as HEV-3 clade 3abchij, three sequences were unclassified to known HEV-3 subtype.

### Material

#### Sample collection

In 2017, a total of 388 rectal swabs were collected from randomly selected pigs (*Sus scrofa domestica*) of different production stages (different age categories) from 25 pig farms located in four districts of Slovakia. These farms housed from ≤100 to ≥1000 animals. The majority of farms focused on two or three production stages of pigs, namely weaners, growers and fatteners. Nine small farms (less than 100 animals) contained weaners and fatteners. Five farms had > 100 animals with sows, suckling piglets and weaners categories. Nine farms were considered as larger units with > 500 pigs of three categories (weaners, growers, fatteners). Only two conventional closed farms had over 1000 pigs with all five production stages (sows, piglets, weaners, growers, fatteners). The health status of all pigs was evaluated by qualified veterinarians on each farm. All pigs were asymptomatic with no clinical signs observed.

Rectal swabs from animals were collected using swab applicators (Sarstedt AG & Co, Germany) with transport medium. The collection of samples from farms is summarized in Table 1. In average, 7–9 samples/farm were collected from suckling piglets (≤ 4 weeks, *n* = 53) weaners (5–10 weeks, *n* = 81), growers (11–16 weeks, *n* = 67), fatteners (≥17 weeks, *n* = 135) and sows (1–3 years, *n* = 52). The collected rectal swabs were delivered to the laboratory at 4 °C within at least 24 h and subsequently processed.

#### HEV RNA extraction and reverse transcription

Rectal swabs were resuspended in 1 ml of 0.01 mol/l PBS (Merck Millipore Corp., USA) for 30 min. This solution was vortexed at 2000 rev. min^− 1^ for 3 min and centrifuged at 14, 000 x g for 5 min. Total RNA was extracted from 140 μl of clarified solution using the QIAamp® viral RNA mini kit (QIAGEN GmbH, Hilden, Germany) by robotic station (QIAcube GmbH, Hilden, Germany) according to the manufacturer’s instruction. The extracted RNA were stored at − 80 °C. The cDNA was synthesized in a 20 μl reaction mix comprising 5 μl of extracted RNA, 0.5 mM dNTPs (Thermo Fisher Scientific, Inc., USA), 200 U RevertAid Premium reverse transcriptase with 1xRT buffer (Thermo Fisher Scientific, Inc., USA), 5 μM of gene specific reverse outer primers [17, 27] (Microsynth Austria, GmbH, Austria), 20 U RNase inhibitor (Invitrogen, Inc., USA) and molecular biology grade water (Merck, GmbH, Germany). The mix was incubated at 65 °C for 5 min then chilled on ice. Subsequently, the mix was incubated at 50 °C for 30 min to synthesise cDNA and finally at 85 °C for 5 min to terminate the reaction.

#### Nested RT-PCR and sequencing

The detection of HEV was based on the amplification of a 287 bp fragment of methyltranspherase (MTase) in ORF1 using outer and inner primers [38] and a 348 bp fragment of capsid protein in ORF2 using primers by Meng et al. [5]. The PCR reaction mix (50 μl) was composed of 1x ThermoPol reaction buffer (New England Biolabs, Inc., USA), 0.2 mM dNTPs (Thermo Fisher Scientific, Inc., USA), 300 nM of outer primers, 1 U Taq DNA polymerase (New England Biolabs, Inc., USA), 4 μl cDNA and molecular biology grade water (Merck, GmbH, Germany). The first PCR was carried out under the following thermal profile: 1 cycle at 95 °C for 1 min, and 35 cycles with denaturation at 95 °C for 30 s, annealing at 55 °C for 1 min, extension at 68 °C for 1 min and final extension at 68 °C for 5 min using Thermocycler C1000 (Bio-Rad Laboratories, Inc., USA). For the second PCR with inner primers an identical thermal profile was used. The size of PCR products was checked by electrophoresis in 2% agarose gel after staining with GelRed™ (Biotium, Inc., USA) and visualized by Gel Doc EZ imager (Bio-Rad Laboratories, Inc., USA). PCR products with the expected size of 287 bp and 348 bp were purified and sequenced by the Sanger method with the PCR primers by a commercial company (Microsynth Austria, GmbH, Austria). PCR products (*n* = 28) for sequencing of ORF1 fragment were randomly selected on the base of DNA amount and electrophoretic quality. For sequencing in ORF2 (*n* = 17), the identical isolates in ORF1 were mostly omitted.

#### Phylogenetic analysis of HEV sequences

Partial ORF1 (242 bp) and ORF2 (304 bp) sequences (primers were omitted) were edited and aligned using the programmes SeqMan, EditSeq and MegAlign (Lasergene, DNASTAR, Inc. USA). Sequences were first checked against the NCBI GenBank database using nucleotide BLAST (http://blast.ncbi.nlm.nih.gov/Blast.cgi).

Two phylogenetic trees were constructed based on the partial ORF1 and ORF2 nucleotide sequences. The molecular evolution model tests and corrected *p-*distance (called shortly *p*-distance in this work) calculations were performed by MEGA6 [39]. Maximum Likelihood phylogenetic analysis of partial ORF1 gene using the General time reversible model with Gamma distribution plus evolutionarily Invariable sites model (GTR + G + I) was used. For the ORF2 gene the Kimura-2 parameter model with Gamma distribution plus evolutionarily Invariable sites model (K2 + G + I) was employed. Models with the lowest BIC scores (Bayesian Information Criterion) were used for phylogenetic analysis. The bootstrap support values of branches were calculated from 1000 replicates. All 45 Slovak HEV nucleotide sequences obtained in this study were submitted to NCBI GenBank database under accession numbers: MT408248–408292.

### Statistical analysis

The differences in detection of HEV among all pig production stages were performed by the chi-square (χ2). Values of *P* < 0.05 were considered statistically significant. The infection prevalence in production stages were evaluated by 95% confidence interval of a proportion. All data analysis were carried out by using GraphPad Prism 5 for Windows (GraphPad Software, Inc. USA).

## Data Availability

The datasets generated and/or analysed during the current study are available in the GenBank repository (MT408248 to MT408292). All data and additional files are available from the corresponding author on reasonable request.

## Abbreviations

HEV: Hepatitis E virus
ORF: Open reading frame
RT-PCR: Reverse transcription - polymerase chain reaction
